# Nutrition for Female Soccer Players—Recommendations

**DOI:** 10.3390/medicina56010028

**Published:** 2020-01-10

**Authors:** Hubert Dobrowolski, Aleksandra Karczemna, Dariusz Włodarek

**Affiliations:** Department of Dietetics, Institute of Human Nutrition Sciences, Warsaw University of Life Sciences (SGGW), 159c Nowoursynowska Str., 02-776 Warsaw, Poland; aleksandra_karczemna@sggw.pl (A.K.);

**Keywords:** athlete, soccer, nutrition, energy needs, nutritional needs, hydration, recommendations

## Abstract

Soccer is one of the most popular sports in the world. As its number of players is increasing, the number of female players is also on the rise. However, there are limited data about how the diets of female soccer players should be designed. Thus, the aim of our work is to deliver concise nutritional recommendations for women practicing this sport. Based on a literature review, we emphasize that individual adjustment of the energy value of the diet is the key factor for the physical performance of female soccer players. Appropriate macronutrient intake makes it possible to achieve the proper energy value of the diet (5–10 g/kg body mass/day carbohydrates; 1.2–1.7 g/kg body mass/day proteins; <30% fats from energy). The micronutrients should be consumed in amounts corresponding to individual values recommended in national standards. Soccer players should pay special attention to the proper consumption of such micronutrients, as well as vitamins such as iron, calcium, and vitamin D. The right amount of fluid intake, consistent with the player’s needs, is crucial in maximizing exercise performance. The diet of a female practicing soccer is usually characterized with low energy values, which increases the risk of various health consequences related to low energy availability. Monitoring the diets of female soccer players is, therefore, necessary.

## 1. Introduction

An optimally balanced diet is one of the factors that positively influences athletic performance. An excess or deficiency of some nutrients may negatively affect sports results [1] and, when optimally adjusted, food rations can ensure maximum body efficiency during training and competition [2]. Many studies have shown that a well-balanced nutritional strategy supports performance in (and recovery from) sporting activities [3]. Therefore, there is a reasonable need to specify dietary recommendations for particular athlete groups to maximize the benefits achieved by training, as well as to increase the chance of success during official competitions.

Soccer is one of the most popular sports in the world and its popularity is still increasing. During 2000–2006, the Fédération Internationale de Football Association (FIFA) estimated that the number of active soccer players increased by 9% (from 242 to 265 million), while the number of active female soccer players over this period increased by 19% (from 21.9 to 26 million) [4]. In 2014, there were 30,145,700 actively training women [5]. FIFA, however, has set one of its goals as increasing the number of women participating in soccer. 

The nutritional needs for women differ from those for men. In numerous nutritional recommendations, separate recommendations for both sexes have been observed. Due to the significant dominance of men in this sport, women have often been marginalized. Among many studies on the nutritional aspects of football players, very few have involved women. There has been little research into the diets of and dietary recommendations for female soccer players.

The aim of our work is to provide concise knowledge and recommendations for female soccer players which can be used by healthcare specialists and soccer trainers, the use of which should maximize the effects of training, increase the chance of achieving sports success, reduce the likelihood of injury, and support the process of post-workout regeneration.

## 2. Energy

Every athlete has individual energy needs, which are indicated by their own sport goals [6]. Therefore, determining energy needs should not only be based on the basic metabolic rate and diet-induced thermogenesis, but also on the individual physical activity of the player; this should be consistent with their individual goals, which will vary over the season, their career, and with sudden injuries and breaks in the training period [7]. It is worth noting that women’s energy needs may be lower due to their lower body weight and less intense training [8].

Several studies have attempted to estimate the energy expenditure of female soccer players during their training. Martin et al. observed an energy expenditure at the level of 2154 ± 596 kcal/day [9]. Fogelhom et al., in turn, showed energy expenditure at the level of 9.42 ± 0.9 MJ/day (~2249 ± 215 kcal/day) [10]. These authors, however, did not compare the obtained results to the context of the player’s body mass. However, their results were different than those in the work of Gibson et al. (2011), where the authors observed energy expenditure at a level of 2546 ± 190 kcal/day, which was calculated according to the current body mass of the study participants, giving 42 ± 3 kcal/kg body mass/day [11]. Our own study (2019) showed energy expenditure among the group of female soccer players at a level of 2811 ± 493 kcal/day (45.7 ± 9 kcal/kg body mass/day) [12]. According to our knowledge, there has been no research directly referring to the energy expenditure of female soccer players during training or official matches. Therefore, it is difficult to relate the obtained values to the actual physical activity related to soccer and non-soccer activity. 

To facilitate the estimation of the energy needs of female soccer players, several papers with recommendations regarding to the diet of female soccer players have been created. FIFA, in its nutrition guide for players, stated that the typical energy expenditure during a match for a 60 kg player is 1100 kcal. In the same guide, in the section on men’s nutrition, FIFA emphasizes, however, that energy expenditure is strongly dependent on the level of competition, football position, style of play, and level of training [13]. However, in our opinion, this information also applies to women. Brewer et al. suggested energy consumption at the level of 47–60 kcal/kg body mass/day is adequate for female soccer players [14]. The Compendium of Physical Activities, last updated in 2011 [15], in turn, stated that the energy cost of a competitive soccer game is 10 METs, while that of a casual game is 7 METs (1 METs = 1 kcal/kg body mass/h). This would mean that, for a player weighing 60 kg, the energy cost for a competitive game would be around 900 kcal, while, for a casual game, this value would be 630 kcal.

It is difficult to relate the energy expenditure measurement results presented in the research to the above recommendations. They relate to the energy expenditure of female players during the game, not their daily needs. Only the research by Gibson et al. [11] showed that energy needs are lower than those recommended in the work of Brewer et al. [14]. In turn, our own research showed that the energy needs were at the lower limit of this standard [12]. This suggests that these recommendations may all have been slightly overestimated.

The dietary energy value, however, is extremely individual. Bloomfield et al. pointed out that players in different field positions devoted different amounts of time to various activities performed at different levels of intensity [16], which certainly results in varied energy expenditure. The purpose of the training itself may significantly vary in intensity and, therefore, in energy expenditure. Finally, trainings can vary significantly during each microcycle, as well as throughout the entire training macrocycle. An individual approach to each player is, therefore, crucial in effectively determining energy needs. Following the recommendations presented in the literature may be connected with the under- or over-estimation of the individual needs of players.

Thus, it is important to properly select and use tools and methods to estimate the total energy expenditure (TEE) of women athletes [3]. TEE can be divided into three main contributions: basal metabolic rate (BMR; 60–80% of TEE), diet-induced thermogenesis (DIT; ~10% TEE), and activity energy expenditure (AEE; ~15–30% TEE) [17,18]. Many methods and tools that can be used to estimate TEE have been introduced. Each of them has their own advantages and disadvantages.

Doubly-labelled water (DLW) or calorimetric methods are considered to be the most accurate methods. This method, using DLW, is based on the assumption that, after taking a dose of double-labelled water consisting of two stable isotopes of deuterium (2H) and oxygen (18O), when the isotopes are included in the total body water pool, they will be removed from the human body at different speeds. Deuterium will be eliminated only in the form of water, while oxygen will be eliminated in the form of both water and CO2. The difference between removing H and O from the body gives a measurement of CO2 production [19,20]. Calorimetric methods can be divided into direct and indirect calorimetry. Direct calorimetry (DC) methods consist of the measuring of the rate of heat loss by the patient’s body, which is carried out in a specially designed, sealed calorimetric chamber. Indirect calorimetry (IC) methods determine energy expenditure in a quantitative manner, by measuring respiratory gases using devices such as Douglas bags, ventilator hoods, and face masks, under specific conditions and, then, by using published formulae. It should be emphasized that IC methods are more accessible than DC and have been increasingly used in clinical settings. In addition, they are practical, safe, noninvasive, and portable, which enhances their attractiveness [17,21,22,23].

The methods described above have the highest accuracy, in terms of estimating athlete’s energy requirements. However, due to their disadvantages (e.g., high cost), prediction equations (PE) have been commonly used to determine TEE. These are readily available and simple-to-use tools for determining the BMR for each individual without any need for specialized equipment. Most often, prediction equations use components such as body mass and height, gender, age, and LBM (lean body mass). Then, to obtain the TEE, the BMR result obtained using PE is multiplied by the factor of physical activity level (PAL). The most commonly used predictive formulas include those given by Harris–Benedict (1919), Mifflin-St. Jeor et al. (1990), Cunningham (1980), Schofield (1985), FAO/WHO/UNU (1985), and Owen et al. (1986–1987) [24]. Unfortunately, despite many advantages, this tool usually shows a tendency to overestimate BMR as well as TEE. In addition, this tool does not account for variables such as ethnic variability, climatic conditions, or nutritional status [24]. 

To determine energy expenditure during physical activity, many noncalorimetric methods (other than DLW) have been used, which are based on extrapolation from various types of variables, measurements (including physiological ones), or observations [25]. One of the most commonly used objective techniques is heart rate monitoring (HRM). The use of HRM relies on the linear relationship between HR and oxygen consumption (VO2) [26]. Unfortunately, many factors can interfere with the performance of the device (e.g., electrical or magnetic interference from common electrical devices [24]). Moreover, the relationship between HR and VO2 differs when taking into account upper-body and lower-body activities; thus, using only one sensor may be associated with obtaining inaccurate results [20,26]. Motion sensors, which include pedometers and accelerometers, have also been used. The function of the former is based on counting the number of steps during walking or running activities. However, these devices are not accurate, as they do not take individual characteristics into account; furthermore, they are vulnerable to manipulation (i.e., shaking the device can increase the number of steps) [26,27,28]. Accelerometers, in turn, are motion sensors that detect acceleration of the body. This acceleration is referred as the rate of change in velocity over a given time. They are characterized by objectivity, noninvasiveness, accuracy, and comfort of use, due to their small size [3]. Unfortunately, they are not very accurate in the case of sedentary activities [29]. In addition, they can affect the participant’s subconscious mind and, thus, increase the amount of physical activity during the study [26]. An additional disadvantage is the often-high cost of the devices. 

Recommendations for energy intake for female soccer players:

- The energy needs should be estimated individually for each athlete, taking into consideration their position and sports goals.
- The energy intake should be periodized with training macro- and micro-cycles, individually adapted with exercise intensity, and aimed at every single training session.
- The energy needs should be estimated using equipment with high measurement accuracy.
- The energy intake should be adjusted to the player’s physical condition and be associated with the optimal lean body mass and low fat percentage.
- The energy value of the diet should not be lower than 30 kcal/kg fat free mass/day, to prevent negative health and performance consequences of low energy availability.

## 3. Macronutrients

Proper intake of proteins, carbohydrates, and fat result in an appropriate energy value of food rations. Moreover, all macronutrients play specific roles in an athlete’s body and, so, their adequate intake is closely related to the maximization of fitness and the chance of success in sports competitions. In Table 1, the current recommendations for macronutrient intake are gathered.

### 3.1. Carbohydrates

Proper carbohydrate intake is a key element in dealing with high training loads among professional athletes [30]. Consumed carbohydrates (CHO), stored as glycogen, are the source of energy for muscles during training [31]. Carbohydrates stored as glycogen in both the liver and skeletal muscles are an essential source of energy, during both matches and training, where the availability of CHO is a limiting factor during long-term physical effort [32]. According to an analysis of soccer matches, the player effort is at the level of 70–80% VO_2_ max; prolonged effort on this level is mostly based on glycogen as a substrate of energy metabolism [33]. Adequate carbohydrate intake before, during, and after training contributes to the maintenance and restoration of glycogen reserves, which will delay the effect of muscle fatigue and improve performance. 

Many sources have indicated the contribution that CHO should have in the energy value of food rations among athletes. However, following these percentages can be misleading. Hassapidou showed, in her work, that this kind of recommendation should be used with caution. A diet with the same energy value can provide a proper or insufficient amount of carbohydrates for athletes with different body masses, when its intake is converted with respect to current body weight [30]. Burke et al. pointed out that, among athletes with high energy needs, carbohydrate consumption may be recommended (according to those sources) at a level of 65%–70%, which may refer to consumption of up to 900 g CHO/day, which exceeds the daily glycogen stores and training costs combined, and the consumption of such amounts can be hard and impractical [34]. Moreover, Burke et al. (2011), in another work, indicated that guidelines connected with the needs of carbohydrates should not be presented in the form of a percentage share of daily energy needs [35].

Recommending carbohydrate intake is much more relevant when taking body mass into consideration. In this way, the recommendations by Academy of Nutrition and Dietetics, Dietitians of Canada, and the American College of Sports Medicine (2016) have been presented. They suggest that, when training is characterized by low intensity or skill-based activities, the CHO needs are 3–5 g CHO/kg body mass/day; for a moderate exercise program (~1 h/day), 5–7 g CHO/kg body mass/day; for an endurance program (1–3 h/day moderate to high-intensity exercise), 6–10 g CHO/kg body mass/day; and an extreme commitment (>4–5 h/day moderate to high-intensity exercise) should be related to the consumption at the level of 8–12 g CHO/kg body mass/day [3]. This position has been supported by the results obtained in numerous studies. In its recommendations, FIFA presented very similar values of CHO needs. The FIFA recommended intakes of 5–7 g CHO/kg body mass/day for moderate duration/low intensity training and 7–10 g CHO/kg body mass/day for moderate-heavy endurance training [13]. Burke et al., in their recommendations for soccer players, recommended 5–7 g CHO/kg body mass/day for moderate daily recovery and match preparation (moderate training program); while, for enhanced daily recovery and match preparation (heavy trainings or two trainings a day) 7–12 g CHO/kg body mass/day was recommended [7]. Focusing on women participating in soccer, The Football Association also recommended 5–7 g CHO/kg body mass/day for a moderate duration/low intensity training program and 7–12 g CHO/kg body mass/day for moderate to heavy endurance training in their recommendations for women [36]. It is worth noting that there is insufficient evidence for differences in the use and storage of glycogen between the sexes. The differences observed in other studies resulted from a lower intake of the energy value of the diet and, thus, from a lower carbohydrate intake [37].

Despite numerous recommendations, an adequate level of CHO intake is rarely achieved. Martin et al., among an examined group of 16 women, observed CHO consumption at the level of 4.1 ± 1.0 g/kg body mass/day [9]. Mullinix et al., in a group of U-21 female soccer players, noticed consumption at a slightly higher level: 4.7 g/kg body mass/day [38]. Clark et al. also obtained comparable results (4.3 ± 1.2 g/kg body mass/day) in a group of female soccer players, when the study was conducted after the football season; however, when the study was conducted before the start of the season, the obtained results were higher (5.2 ± 1.1 g/kg body mass/day) [39]. Gibson et al. observed a consumption of 5 ± 1.6 g/kg body mass/day [11]. Our own study has shown that female soccer players had a carbohydrate intake of 3.28 ± 1.2 g/kg body mass/day [12]. Thus, according to the literature, insufficiency or consumption being at the lower limit of recommendations has often been observed. Low CHO consumption may be a result of low energy content in the diet. However, failure to meet the minimum CHO recommended intake is almost certainly related to a lack of adequate glycogen resynthesis.

In addition to the adequate daily CHO intake, it is also important to provide the right amount of macronutrients at specific workout-related times. The first of these periods is during the time before physical activity. Carbohydrate intake over a certain period of time before training or a match increases glycogen stores, stabilizes blood sugar levels, and saves muscle and liver glycogen. It has been indicated that, with activity lasting above 90 min, carbohydrate loading of 10–12 g CHO/kg body mass/day every 24 h for a period of 36–48 h is an effective dose [35]. Pre-event fueling has been connecting with taking a dose of 1–4 g CHO/kg body mass/day 1–4 h before training [35]. Lee et al. noticed that the intake of carbohydrates in female athletes before starting physical activity contributed to increasing the effectiveness of repeated sprints [40]. Hassapidou suggested that 200–300 g of CHO consumed 3–4 h before activity may be beneficial [30]. However, this is a risky statement, as such a high dose for competitors with lower body weight (as in the case of women) may be too high. Excessive doses of carbohydrates do not necessarily increase the level of CHO oxidation and can cause gastrointestinal problems [41]. When planning pre-training carbohydrate intake, attention should also be paid to training frequency. It is impossible to conduct carbohydrate loading with daily trainings or competitions. Therefore, it is recommended that this form of carbohydrate supply takes place before major training and matches, while milder carbohydrate intake methods should be considered for daily training.

Another specific period for carbohydrate intake is the time of training and competition. The consumption of carbohydrates during exercise improves performance by maintaining blood glucose levels and maintaining CHO oxidation at a high level, saving endogenous glycogen stores or synthesizing glycogen during training at a low intensity [42]. The importance of carbohydrate intake during exercise has been emphasized in the literature. Ali et al. noted that carbohydrate intake during exercise allowed players to maintain football skills and sprints longer, compared to the placebo group [33]. Russel et al. observed that intake of CHO reduced the decrease in performance during a simulated game [43]. However, these studies were conducted with men. There have been no comparable studies in the female soccer players group, but there has also been no evidence that the consumption of carbohydrates during exercise has a different effect for males. Jeunkendrup (2014) recommended that the amount of CHO intake during a 1–2 h physical effort (e.g., standard training or football match) should be 30 g CHO/h of training, while 2–3 h of effort corresponded to 60 g CHO/h. In his work, he also emphasized that, in team sports and sports requiring specific skills, the intake of carbohydrates not only delays the effect of fatigue, but also improves the skills associated with the sport, especially during the final stages of the game [44]. Burke et al., in previous recommendations, also mentioned almost identical values: 30–60 g CHO/h for effort lasting 1–2.5 h [35].

Finally, an important element is the adequate intake of carbohydrates after exercise. Glycogen reserves are reduced during training or sporting events. CHO supply after physical activity is intended for glycogen resynthesis. Studies have shown that 20–24 h glycogen resynthesis is able to normalize glycogen levels after depletion during exercise, in the case of a recovery rate at the level of 5% every hour [45]. However, it should be emphasized that most trainings will not result in the full use of glycogen stores and, so, glycogen resynthesis will end much earlier. Of course, the daily recommendations for carbohydrate intake have also been determined on the basis of the need for glycogen resynthesis; therefore, with a normal training plan, additional carbohydrate amounts are not necessary. However, if two separate trainings are preceded by a short break (e.g., two workouts during one day or the first training taking places during evening one day and the second during the morning of the next day), maximum glycogen resynthesis should be ensured. The fastest rate of glycogen recovery takes place in the first few hours after training, which is caused by a series of factors such as activation of glycogen synthase, increased insulin sensitivity caused by physical activity, and muscle cell membrane permeability for glucose [46]. Hence, it is advisable to supply carbohydrates as soon as possible after a workout. Recommendations have indicated a dose of 1–1.2 g/kg body mass/h for the first 4 h, when there are less than 8 h between two training sessions [35]. It should be noted, however, that not all studies involving female soccer players have confirmed the indicated rate of glycogen recovery. Krustrup et al. noticed that, 24 h after a football match, muscle glycogen levels were 27% lower than in the control group, despite a high carbohydrate diet (9.5 g CHO/kg body mass/day) [47]. However, these studies were carried out on men with higher muscle masses than women. Soccer matches among women are also less demanding than for men [48]. This suggests, therefore, that the recommended values should apply to women participating in soccer.

### 3.2. Proteins

Proteins play an important role in an athlete’s diet, taking part in the regulation of muscle protein synthesis, weight regulation, growth stimulation, and post-workout regeneration, among others. Among team sports athletes, proteins and amino acids are perceived as crucial for performance [49]. Despite reports suggesting comparable protein needs in athletes and less active adults, there have been many studies indicating a significantly higher protein demand among athletes [50]. The International Society of Sports Nutrition stated, in its guidelines, that the majority of physically active people should consume 1.4–2.0 g/kg body mass/day to optimize exercise training-induced adaptations [51]. According to the recommendations of Academy of Nutrition and Dietetics, Dietitians of Canada, and the American College of Sports Medicine, optimal protein intake is in the range of 1.2–2.0 g/kg body mass/day, with higher doses being required for short-term training intensification and/or reducing energy intake with diet [3]. Referring to the needs of football players, Lemon emphasized that protein intake at the level of 1.4–1.7 g/kg body mass/day should be adequate [52]. Tipton and Wolfe, in turn, concluded that the recommended protein intake for athletes should be in the range of 1.2–1.7 g/kg body mass/day [49]. In her work, Dolins indicated that, for physically active women, a dose of 1.2 g of protein/kg body mass/day is sufficient to maximize sporting achievements [53]. Boisseau et al. stated that the Estimated Average Requirement (EAR) for football players should be 1.2 g/kg body mass/day, while the Recommended Dietary Allowances (RDA) is 1.4 g/kg body mass/day [54]. Although most studies and recommendations have referred to men or are targeted at athletes as a whole, protein recommendations are adequate for both men and women who train football [55].

In most cases, studies have shown that female soccer players consume adequate amounts of protein. Martin et al. observed consumption at the level of 1.2 ± 0.3 g/kg body mass/day [9]. Mullinix et al., in turn, noticed protein consumption at the level of 1.3 g/kg body mass/day [38]. Clark et al., in their work on the assessment of protein content in the food ration of female football players before the start of the football season, noticed intake in line with the demand of 1.4 ± 0.3 g/kg body mass/day; however, after the end of the football season, the study gave significantly worse results: 0.96 ± 0.3 g/kg body mass/day [39]. Consumption in accordance with the recommendations was also noted by Gibson et al., who showed a consumption of 1.4 ± 0.3 g/kg body mass/day [11]. Our own research showed protein intake among football players to be slightly below the recommended values: 1.18 ± 0.44 g/kg body mass/day [12]. As the vast majority of training football women consume protein in amounts lower than 1.4 g/kg body mass/day without a decrease in efficiency, it can be concluded that the lower limit of 1.2 g/kg body mass/day indicated in the recommendations is appropriate for this group.

As in the case of carbohydrates, protein should also be consumed at appropriate times around training. The main period of special demand is the time after training. Protein, as a stimulant of regenerative processes, regulates muscle protein synthesis and inhibits protein breakdown. Studies have shown that an intake of 20 g of proteins or 9 g of EEA stimulates the process of muscle protein synthesis during and up to 2 h after exercise [56]. In addition, as showed by Levenhagen et al., eating a meal immediately after physical activity results in better regenerative effects than when eating the same meal 3 h after the end of activity [57]. Protein consumption right after training is, therefore, crucial for regenerative processes. Additionally, the intake of protein after exercise (in the amount of 0.2–0.4 g/kg body mass/h), together with carbohydrates (0.8 g/kg body mass/h) accelerated glycogen resynthesis. However, these correlations were not observed when the post-workout supply of carbohydrates exceeded a dose of 1.2 g/kg body mass/h [56]. It is recommended to consume 0.3 g protein/kg body mass after key training sessions and every subsequent 3–5 h, in the form of many meals, to increase muscle adaptation to training [3]. Unfortunately, at present, most studies concerned with post-workout muscle regeneration and the effects of post-workout protein supply on muscle strength have been carried out in relation to resistance training, and those regarding team sports are very few. According to our knowledge, there have been no studies related to female football teams.

Special attention should be paid, however, to leucine. It is one of the three branched-chained amino acids (along with isoleucine and valine), which constitute approximately one-third of skeletal muscle proteins [58]. Many observations of the leucine threshold for the activation of muscle protein synthesis have been made [59]. Studies have shown that leucine has a controlling influence over the activation of muscle protein synthesis [60]. Adequate leucine supply is, therefore, significant for stimulating muscle protein synthesis. Mero suggested that the RDA for leucine is minimum 45 mg/kg body mass/day for sedentary individuals, with a higher amount for those participating in intensive training [58]. This value can be easily achieved by consuming the minimum requirement of 1.2 g proteins/kg body mass/day. In their recent recommendation, the International Society of Sports Nutrition suggested that a protein dose of 20–40 g of protein (10–12 g of EAAs, 1–3 g of leucine) stimulates MPS, making high-quality protein sources that are rich in EAAs and leucine preferred sources of proteins. Athletes should focus on consuming adequate leucine content in each of their meals through selection of high-quality protein sources. Such doses of protein containing 700–3000 mg of leucine should be consumed every 3–4 h, spread across the day [51].

Therefore, protein sources play a key role in athletic nutrition. Only high-quality proteins supply appropriate amounts of EAA and leucine. Animal sources typically possess a higher biological value than vegetable sources, due to vegetable sources lacking one or more of the essential amino acids [61]. High-quality proteins, indexed in terms of protein digestibility corrected amino acid score (PDCAAS) are milk, eggs, and most meats [62]. Protein consumption from these sources is beneficial in terms of EAAs and leucine. Vegetarians have a more difficult task, as vegetarian diets often lack equivalent amounts of proteins when compared to omnivorous diets [63]. A good solution to this is soy proteins: Isolated soy protein, once the antinutritional components are removed, have PDCAAS scores comparable to animal-source proteins. Moreover, soy is a complete protein with high concentrations of BCAAs [61].

### 3.3. Fats

Fats, apart from additional energy source, are an essential element of cell walls and are a source of fat-soluble vitamins, including vitamin D, which is one of the nutrients of particular interest. However, despite the role of the fat in human body, carbohydrates and proteins play much more important roles in an athlete’s diet; hence, female football players should first cover the demands of these macronutrients, while fats should supplement the energy value of the food ration. Clark emphasized that fats should account for less than 30% of the energy value of a player’s diet [8]. However, it is recommended that athletes do not consume less than 20% of the dietary energy value in the form of fat for an extended period, as this may lead to a reduction in the consumption of fat-soluble vitamins and essential fatty acids [3]. Evidence of the advantage of a high-carbohydrate diet over a high-fat diet for football players has been provided in a study by Souglis et al. (2013) [64]. They observed that players who used a high carbohydrate diet (565 g CHO/day and 44 g fat/day) for 3.5 days covered a greater distance during the match, compared to a low carbohydrate diet (212 g CHO/day and 186 g fat/day).

## 4. Micronutrients

To our knowledge, there have been no studies indicating the actual micronutrient demands among athletes. It is assumed that these needs are actually higher than those suggested for physically inactive people. Athletes, however, consume much more food due to greater energy needs. These diets, due to the higher energy value, also contain larger amounts of micronutrients. Therefore, it is recommended for athletes to consume amounts of micronutrients at levels referring to the RDA and Adequate Intake (AI), at least [3]. However, due to the special roles that some minerals and vitamins play in an athlete’s diet, we describe the micronutrients to which football players should pay special attention.

### 4.1. Iron

Iron is one of the key micronutrients in an athlete’s diet. In the human body, it is responsible for oxygen transport and energy production [65], and its adequate intake through diet, absorption, and cellular use is crucial for endurance performance [66]. Anaemia, caused by iron deficiency, can result in decrease of performance; furthermore, depletion of iron stores affects aerobic training adaptation, increases muscle fatigue, and decreases energetic efficiency during submaximal exercise [67]. However, athletes, as a group, are particularly vulnerable to iron deficiency, because of its loss due to gastrointestinal bleeding (often observed among athletes), exercise-induced haematuria, and loss of iron along with sweat that is produced during training, as well as training itself [68].

Women are especially vulnerable to iron deficiency among athletes [67,68,69]. This is caused, among other reasons, by the loss of this micronutrient during period [68]. It is worth noting that the demand for iron among female athletes can be increased by up to 70% EAR [3]. DellaValle pointed out that there is a need for supervision of haemoglobin and ferritin levels in women athletes at risk of iron deficiency, both at the beginning of and during the training period. She also drew attention to the need for appropriate recommendations regarding iron intake and supplementation among athletes who have been diagnosed with its deficiency [70]. In turn, Landahl et al. showed that iron deficiency is a common problem among female soccer players [71].

### 4.2. Calcium and Vitamin D

Calcium and vitamin D are key nutrients for athletes, mainly due to their roles in maintaining bone health. Their insufficient delivery to the body can cause bone loss and lead to a risk of bone damage and injury. In addition, calcium is a mineral that plays an important role in the bodies of physically active women: it takes part in blood clotting, muscle contractions, nerve transmission, protein utilization, and cellular communication [72]. Vitamin D and its impact on athletic performance, in turn, has aroused more and more interest among researchers. Although studies have shown that vitamin D has an impact on physical performance among athletes who have been diagnosed with its deficiency [73], a meta-analysis of randomized control studies by Farrokhyar et al. did not show that vitamin D significantly increased physical performance [74].

Athletes, especially women, are particularly at risk of calcium and vitamin D deficiencies and their negative consequences. Studies have shown that young women practicing vigorous exercises are at risk of inhibiting bone mineral growth [75]. Another reason for lower bone mineral density may be low energy availability or menstrual dysfunction, which is described later in this work. According to the literature, vitamin D deficiencies can affect 33%–42% of female athletes [76], although, in football, these deficiencies are observed less frequently. This may be the result of exposure to sunlight: vitamin D is produced endogenously in the skin due to UVB radiation, to which players are exposed during outdoor training. 

The consequence of calcium and vitamin D deficiency is the loss of bone mass and an increased risk of fracture. In addition to the possible permanent damage to health and an inability to return to regular training, there is certainly temporary exclusion from physical activity. This will result in a significant drop of the player’s condition and a lack of possibility for achieving any success in the competition, for a certain period. This, in turn, will affect the entire professional career of the athlete and other aspects of their life (e.g., economic aspects). It is, therefore, important to minimize the risk of such deficiencies and consequent injury. As indicated by studies involving female navy recruits, supplementation with vitamin D (800 UI/day) and calcium (2000 mg/day) reduced the risk of fractures by as much as 20% [77].

## 5. Hydration

Optimal hydration is one of the main factors determining the effectiveness of playing (on the pitch) during training and sports competitions. In addition, providing the right amount of fluids both before, during, and after training can affect an athlete’s performance. According to studies, dehydration adversely affects muscle strength, endurance, motor coordination, mental performance, and thermoregulation processes [78]. Therefore, maintaining an optimum level of hydration seems crucial for athletic performance. Dehydration during training at a level of 1–2% of body weight compared to the initial weight before physical activity, may result in deterioration of exercise capacity among footballers [79].

Despite the great importance of optimal hydration for exercise capacity, athletes are often dehydrated. Casro-Sepulveda et al., in their study of a group of female football players, showed that only one among 17 players was hydrated and two were minimally dehydrated [80]. Gibson et al. indicated that 45.4% of female players were dehydrated before training [81]. Different results were presented by Kiding et al., who mostly studied adequate pre-workout hydration in a group of footballers [82].

Weight loss due to dehydration among footballers has been commonly observed. Gibson et al. reported a decrease in body weight of less than 1%, which was similar to the aforementioned studies by Kiding et al. [81,82]. Chaud et al. observed dehydration among female football players during training at a comparatively high level [83]. However, women’s sweat loss during soccer training is much lower, compared to men. A lower level of dehydration during training (<1% body mass) was shown by Philips et al. [84]; however, other authors observed weight loss higher than 1.5% of body weight [85,86].

These differences may be due to differences in body size. As Rossi pointed out in her work, lower sweat loss among women compared to men is a known and studied fact, which has been associated with a smaller body size and a greater ratio of body surface area to body mass, which results in less sweating by up to 34% [74]; hence, the needs of female athletes for fluids are slightly lower compared to men.

To maintain optimal physical performance during physical activity, the euhydrated state should be first achieved. The American College of Sports Medicine recommends drinking 5–7 mL/kg body weight four hours before training and an additional 3–5 mL/kg body weight two hours before starting exercises in the absence of urine or very dark urine (which indicates significant dehydration) [87].

Hydration during training depends on the type and intensity of the physical effort: whether it is just training or a match. While regular hydration is possible during training, during official matches it is limited to half-time breaks and minor breaks (e.g., as a result of a serious foul by one of the players). The level of sweat loss (and, therefore, the rate of dehydration) also depends on several environmental conditions, such as temperature or humidity. It has been estimated that the rate of sweat loss during exercise can range from 0.3–2.4 L/h [3]. Therefore, it is difficult to assess the exact needs for female football players, especially when the rate of sweat loss is lower than that of men. A good solution is to frequently drink small volume of liquids, in order to not fill the stomach, which provides a cause of discomfort and may reduce the player’s performance. 

During training, players should drink fluids at a rate that prevents any weight loss greater than 2%. Individual assessment of fluid loss in the form of periodic measurement of body weight before and after training, along with individual adjustment of the hydration plan, seems to be the best solution [87].

An additional step to maintaining optimal hydration is adequate fluid intake after physical activity. FIFA recommends drinking 1.2–1.5 L of fluid for every kilogram of body weight lost during training or a match [13]. Similar recommendations (1.5 L for each 1 kg body weight loss) have been proposed by ACSM [87]. Fluid intake at this level will allow optimal hydration and accelerate the regeneration process.

It should also be noted that, beyond water, sweat also contains other important ingredients—electrolytes—that are lost. It has been estimated that the concentration of sodium in sweat is ~35 mEq/L, potassium 5 mEq/L, calcium 1 mEq/L, magnesium 0.8 mEq/L, and chlorine 30 mEq/L [87]. Although women, as in the case of water, lose less electrolytes than men, it is recommended to intake drinks containing electrolytes during and after training to compensate for their loss with sweat [87]. It is also possible to add carbohydrates to drinks consumed during training, in order to maximize the amount of energy needed for physical effort (the optimal dose of carbohydrates during training was mentioned in an earlier part of the work). In addition, it should be noted that the presence of carbohydrates and proteins in drinks may increase fluid absorption in the intestine [88,89].

Recommendations are summarized in Table 2.

## 6. Female Athlete Triad and Relative Energy Deficiency in Sport (RED-S)

The female athlete triad (the triad) is a term referring to three coexisting and dependent components: low energy availability, menstrual disorders, and low bone mineral density. These three disorders may occur separately or together, and negatively affect the health and performance of both professional and amateur female athletes [90]. Although many authors have assumed that one of the triad components is eating disorders, which results in low energy availability, this is a mistaken assumption, as low energy consumption (and hence low energy availability) may be the result of other factors, such as a lack of proper education or weight reduction without proper nutritional care.

The development of the triad begins with low energy availability, which arises as a result of limited food consumption in relation to increased demand. The reason for low consumption may be eating disorders or the aim to reduce body weight. Low energy availability can also arise due to an increase in the volume or intensity of training while not increasing the energy value of the athlete’s food ration. Low energy availability leads to menstrual disorders by inappropriate luteinizing hormone (LH) secretion that, in turn, leads to the disorder of gonadotropin-releasing hormone (GnRH) secretion. Ultimately, this causes functional hypothalamic amonorrhea. Menstrual disorders, in turn, lead to estrogen deficiencies, which limit skeletal formation and increase bone resorption. In addition, low energy availability causes increased bone resorption due to the impaired action of other hormones, such as cortisol or leptin. Disorders of LH secretion and bone health have been observed when energy availability fell below 30 kcal/kg fat free mass/day [91].

In 2014, however, the International Olympic Committee (IOC) published a paper in which it expanded the phenomenon of disorders resulting from low energy availability; they called this spectrum of occurring irregularities as the Relative Energy Deficiency in Sport (RED-S) [92]. As IOC mentioned, relatively low energy availability can cause a number of health disorders—including impairment of immunological functions; menstrual-function disorders; osteoporosis; disorders of the endocrine, metabolic, haematological, psychological, gastrointestinal, and cardiovascular systems; and growth disorders—and affect exercise capacity (e.g., increased injury risk, decreased training response, impaired judgment, decreased motor co-ordination and concentration, irritability, depression, decreased glycogen supplies, and decreased muscle strength and stamina) [92,93].

Adequate energy intake with diet is, therefore, crucial for athletes. However, reported results of consumption among female football players are at a critically low level. Martin et al. observed energy consumption at the level of 30.9 ± 5.5 kcal/kg body mass/day [9]. Gibson et al. reported consumption at the level of 35 ± 10 kcal/kg/day [11]. Clark et al. (2013) also noticed low energy consumption: during the football season, the examined group of female players consumed 37.0 ± 5.0 kcal/kg/day while, after the end of the season, they observed 30 ± 18.0 kcal/kg/day [39]. In our own work, we recorded consumption at the level of 24.3 ± 8.9 kcal/kg/day [12]. The authors of other studies did not scale energy consumption to the player’s body weight, but they reported comparatively low values in the context of daily consumption [10,38,94].

It is noteworthy that disorders associated with the female athlete triad and RED-S have been observed when the amount of available energy was lower than 30 kcal/kg fat-free mass/day. The low energy consumption among female football players observed in the literature referred to kilograms of current body weight, not lean body mass. Thus, if the above studies showed an average consumption equal to or slightly higher than 30 kcal/kg body weight/day, then there were certainly some women in the study groups whose energy intake from the diet was less than 30 kcal/kg fat-free mass/day. Therefore, there is reasonable suspicion that female football players are a group at risk of RED-S. However, to our knowledge, there have been no studies that directly examined the frequency of this syndrome in a group of women participating in football.

## 7. Limitations

There are some limitations to the dietary recommendations for female soccer players presented in our paper. The major limitation, when it comes to providing concise recommendations for women practicing soccer, is the lack of studies that have taken female soccer players as a study group. There have only been a few studies indicating what their diet should actually look like and only single ones indicating the amounts of particular nutrients that should be consumed. Most recommendations, interventions, and trials have been conducted on male soccer players. Some studies have compared the nutritional needs of men and women; by taking their results into consideration, we can conclude what female soccer players’ diets should look like, along with their nutritional needs. Studies conducted with female soccer players, as well as other team sports female athletes, therefore, are urgently necessary.

## 8. Conclusions

In summary, female soccer players are a group that has specific nutritional needs. The energy value of the diet should be determined for each player individually, as these needs can be extremely different between players; even those with similar anthropometric parameters. Adequate intake of carbohydrates (5–10 g/kg/day), proteins (1.2–1.7 g/kg/day), and fats (<30% of energy from the diet), as well as macronutrients, during training periods will allow players to achieve appropriate dietary energy values, as well as maximizing their physical performance and accelerating the regeneration process after training. Adequate amounts of micronutrients, in accordance with current demands and individually adjusted to the needs of fluid intake, will also affect the appropriate health conditions and maximize the exercise capacity of football players. Adequate consumption, in terms of energy and intake of macronutrients, is also crucial for health issues among the female football players group. Due to the low energy consumption frequently observed in the literature, the diets of female football players should be periodically monitored and corrected to prevent the occurrence of abnormal eating behaviors and the corresponding health consequences, as well as to support their performance. However, there has been no research specifically related to the needs of women who play football. Most recommendations for this group have been based on recommendations for men. More studies connecting to the nutritional needs of women practicing this sport and the risks of adverse effects due to an improper diet are required.

## Figures and Tables

**Table 1:** Recommendations for macronutrient intake for female soccer players.

Macronutrient	General	Before Training	During Training	After Training
Carbohydrates	5–7 g CHO/kg body mass/day with low to moderate intensity training program. 7–12 g CHO/kg body mass/day with high intensity training program or match preparation.	1–4 g CHO/kg body mass for 1–4 h before training 10–12 g CHO/kg body mass/day for period of 36–48 h before major competition.	30 g CHO/h for training lasting 1–2 h.	1–1.2 g CHO/kg body mass/h for first 4 h (if there are less than 8 h between training sessions).
Proteins	1.2–1.7 g/kg body mass/ day 20–40 g of proteins (containing 700–3000 mg leucine) every 3–4 h to maximize MPS.			20 g of proteins or 9 g of EEA during and up to 2 h after training. 0.3 g of proteins/kg body mass after training and every subsequent 3–5 h.
Fat	Less than 30% of the energy value of the diet. Not less than 20% of the dietary energy value to prevent deficiency of fat-soluble vitamins and essential fatty acids.			

**Table 2:** Recommendation for fluid intake for female soccer players.

Timing	Recommendation
Before training	Try to prevent dehydration before training by drinking fluids over the day. 5–7 mL/kg body weight 4 h before training. Additional 3–5 mL/kg body weight 2 h before starting exercises in the absence of urine or very dark urine.
During training	Drink fluids to avoid 2% dehydration level. Drink in small portions to not overfill stomach.
After training	Drink 1.5 L of fluids for each 1 kg body mass loss. Addition of electrolytes can replace those lost with sweat. Presence of carbohydrates and proteins in drinks may increase intestinal fluid absorption.
